# First-line options for systemic juvenile idiopathic arthritis treatment: an observational study of Childhood Arthritis and Rheumatology Research Alliance Consensus Treatment Plans

**DOI:** 10.1186/s12969-022-00768-6

**Published:** 2022-12-08

**Authors:** Timothy Beukelman, George Tomlinson, Peter A. Nigrovic, Anne Dennos, Vincent Del Gaizo, Marian Jelinek, Mary Ellen Riordan, Laura E. Schanberg, Shalini Mohan, Erin Pfeifer, Yukiko Kimura, for the CARRA FROST Investigators, R. Agbayani, S. Akoghlanian, E. Allenspach, E. Anderson, S. Ardoin, S. Armendariz, I. Balboni, L. Ballenger, S. Ballinger, F. Barbar-Smiley, K. Baszis, H. Bell-Brunson, H. Benham, W. Bernal, T. Bigley, B. Binstadt, M. Blakley, J. Bohnsack, A. Brown, M. Buckley, D. Bullock, B. Cameron, S. Canna, E. Cassidy, J. Chang, V. Chauhan, T. Chinn, P. Chira, A. Cooper, J. Cooper, C. Correll, L. Curiel-Duran, M. Curry, A. Dalrymple, D. De Ranieri, F. Dedeoglu, M. DeGuzman, N. Delnay, V. Dempsey, J. Dowling, J. Drew, K. Driest, Q. Du, D. Durkee, M. Eckert, C. Edens, M. Elder, S. Fadrhonc, L. Favier, B. Feldman, I. Ferguson, B. Ferreira, L. Fogel, E. Fox, R. Fuhlbrigge, J. Fuller, N. George, D. Gerstbacher, M. Gillispie-Taylor, I. Goh, D. Goldsmith, S. Grevich, T. Griffin, M. Guevara, P. Guittar, M. Hager, T. Hahn, O. Halyabar, M. Hance, S. Haro, J. Harris, J. Hausmann, K. Hayward, L. Henderson, A. Hersh, S. Hillyer, L. Hiraki, M. Hiskey, P. Hobday, C. Hoffart, M. Holland, M. Hollander, M. Horwitz, J. Hsu, A. Huber, M. Ibarra, C. Inman, S. Jackson, K. James, G. Janow, S. Jones, K. Jones, J. Jones, C. Justice, U. Khalsa, B. Kienzle, S. Kim, Y. Kimura, M. Kitcharoensakkul, T. Klausmeier, K. Klein, M. Klein-Gitelman, S. Kramer, J. Lai, B. Lang, S. Lapidus, E. Lawson, R. Laxer, P. Lee, T. Lee, M. Lerman, D. Levy, S. Li, C. Lin, N. Ling, M. Lo, S. Lvovich, J. Maller, A. Martyniuk, K. McConnell, I. McHale, E. Meidan, E. Mellins, M. Miller, R. Modica, K. Moore, T. Moussa, V. Mruk, E. Muscal, K. Nanda, L. Nassi, J. Neely, L. Newhall, P. Nigrovic, B. Nolan, E. Oberle, O. Okeke, M. Oliver, K. O’Neil, R. Oz, A. Paller, J. Patel, P. Pepmueller, K. Phillippi, R. Pooni, S. Protopapas, B. Puplava, S. Radhakrishna, S. Ramsey, H. Reid, S. Ringold, M. Riordan, M. Riskalla, M. Ritter, M. Rodriquez, K. Rojas, M. Rosenkranz, T. Rubinstein, C. Sandborg, L. Scalzi, K. Schikler, K. Schmidt, E. Schmitt, R. Schneider, C. Seper, J. Shalen, R. Sheets, S. Shenoi, J. Shirley, E. Silverman, V. Sivaraman, C. Smith, J. Soep, M. Son, L. Spiegel, H. Stapp, S. Stern, A. Stevens, B. Stevens, K. Stewart, E. Stringer, R. Sundel, M. Sutter, R. Syed, R. Syed, T. Tanner, G. Tarshish, S. Tarvin, M. Tesher, A. Thatayatikom, B. Thomas, D. Toib, K. Torok, C. Toruner, S. Tse, T. Valcarcel, N. Vasquez, R. Vehe, J. Velez, E. von Scheven, S. Vora, L. Wagner-Weiner, D. Wahezi, M. Waterfield, P. Weiss, J. Weiss, A. White, L. Woolnough, T. Wright, M. Yee, R. Yeung, K. Yomogida, Y. Zhao, A. Zhu

**Affiliations:** 1 University of Alabama at Birmingham, 1601 4th Ave South, CPPN G10, Birmingham, AL 35233 USA; 2 Institute of Health Policy, Management and Evaluation, Dalla Lana School of Public Health, University of Toronto, Toronto, ON Canada; 3 Division of Immunology, Boston Children’s Hospital, Boston, MA 02115 USA; 4 Division of Rheumatology, Inflammation, and Immunity, Brigham and Women’s Hospital, Boston, MA 02115 USA; 5 Duke Clinical Research Institute, Duke University, Durham, NC 27715 USA; 6 Childhood Arthritis and Rheumatology Research Alliance, Washington, DC USA; 7 Joseph M Sanzari Children’s Hospital, Hackensack Meridian School of Medicine, Nutley, NJ 07110 USA; 8 Department of Pediatrics, Duke University School of Medicine, Durham, NC 27710 USA; 9 Genentech Inc., South San Francisco, CA 94080 USA

**Keywords:** Systemic juvenile idiopathic arthritis, Juvenile idiopathic arthritis, Still’s disease, Treatment, Biologics

## Abstract

**Background:**

The Childhood Arthritis and Rheumatology Research Alliance (CARRA) developed consensus treatment plans (CTPs) to compare treatment initiation strategies for systemic juvenile idiopathic arthritis (sJIA). First-line options for sJIA treatment (FROST) was a prospective observational study to assess CTP outcomes using the CARRA Registry.

**Methods:**

Patients with new-onset sJIA were enrolled if they received initial treatment according to the biologic CTPs (IL-1 or IL-6 inhibitor) or non-biologic CTPs (glucocorticoid (GC) monotherapy or methotrexate). CTPs could be used with or without systemic GC. Primary outcome was achievement of clinical inactive disease (CID) at 9 months without current use of GC. Due to the small numbers of patients in the non-biologic CTPs, no statistical comparisons were made between the CTPs.

**Results:**

Seventy-three patients were enrolled: 63 (86%) in the biologic CTPs and 10 (14%) in the non-biologic CTPs. CTP choice appeared to be strongly influenced by physician preference. During the first month of follow-up, oral GC use was observed in 54% of biologic CTP patients and 90% of non-biologic CTPs patients. Five (50%) non-biologic CTP patients subsequently received biologics within 4 months of follow-up. Overall, 30/53 (57%) of patients achieved CID at 9 months without current GC use.

**Conclusion:**

Nearly all patients received treatment with biologics during the study period, and 46% of biologic CTP patients did not receive oral GC within the first month of treatment. The majority of patients had favorable short-term clinical outcomes. Increased use of biologics and decreased use of GC may lead to improved outcomes in sJIA.

## Background

Systemic Juvenile Idiopathic Arthritis (sJIA) is characterized by systemic inflammation that distinguishes it from other types of JIA. sJIA can have life-threatening complications, including macrophage activation syndrome (MAS) which can occur at any time during the disease. In North America/Europe, sJIA is a rare disease, and accounts for 5 to 15% of children with JIA. Age at onset is often in early childhood, with a peak from 1 to 5 years of age, but sJIA can develop at any age, and after the age of 16 is called Adult Onset Still Disease (AOSD) [1].

Prior to the availability of biologic medications, treatment of sJIA was difficult, often requiring prolonged courses of systemic glucocorticoids (GC), which cause many adverse effects including growth failure, osteoporosis, and infections. Major advances in the treatment of sJIA began with reports of the effectiveness of the IL-1 inhibitor (IL-1i) anakinra in the mid 2000s [2–4]. Results of controlled trials of canakinumab and rilonacept confirmed the efficacy of IL-1i after 2010 [5, 6]. The IL-6 inhibitor (IL-6i) tocilizumab proved to be equally efficacious [7]. Since those studies, IL-1i and IL-6i have been increasingly used for treating sJIA, along with GC and methotrexate (MTX). A hypothesis has emerged that the use of biologics (especially IL-1i and potentially IL-6i) early in the disease course may allow patients a window of opportunity to prevent the evolution of chronic, destructive synovitis [8]. This was suggested by an early retrospective case series as well as a recent prospective study in which patients with sJIA were initially treated with anakinra alone [9]. Interestingly, the aforementioned published randomized clinical trials enrolled patients with long-standing (often refractory) sJIA, and so did not provide information about which treatments are most effective for patients with new-onset sJIA [5, 7]. As a result, there continues to be uncertainty about treatment choice at the time of sJIA diagnosis. This uncertainty is compounded by continued reports of rare cases of chronic lung disease which appear temporally associated with increased use of biologic agents in sJIA [10–13].

To help answer these important questions, the Childhood Arthritis and Rheumatology Research Alliance (CARRA) developed four consensus treatment plans (CTPs) for new-onset sJIA in 2012 based on the initial treatments most commonly used at the time. The CTPs were: (1) GC alone, (2) MTX, (3) IL-1i (anakinra or canakinumab), and (4) IL-6i (tocilizumab), each of which could be used with or without GC [14]. The CTPs were developed as standardized consensus-based treatments which were intended to be used for observational comparative effectiveness research using the CARRA Registry as the data collection vehicle [15]. A pilot study of the sJIA CTPs was completed in 2016 and showed good distribution of CTPs used among the 13 sites that enrolled patients, making a larger comparative effectiveness study feasible [16]. This approach was also successfully used in the recently published Start Time Optimization of biologics in Polyarticular JIA (STOP-JIA) study [17, 18].

The FiRst-line Options for SJIA Treatment (FROST) study was intended to be an observational comparative effectiveness study of the sJIA CARRA CTPs enrolling new-onset sJIA patients with data collected in the CARRA Registry. Herein we report the primary results of the study.

## Methods

The CARRA JIA Research Committee prioritized sJIA as one of 4 initial diseases to develop CTPs for comparative effectiveness research using the Registry funded by an NIH ARRA Challenge Grant. A group of sJIA experts worked together to identify current treatments most commonly used for sJIA. Through a process of surveys, face-to-face consensus meetings and small workgroup conference calls, leaders developed the four CTPs which were finalized and approved by 95% of the CARRA JIA Research Committee and published in 2012 [14]. To better conform with the diagnostic approach to sJIA in clinical practice, the CARRA JIA Research Committee also voted to modify the sJIA ILAR criteria [19] for this study. Eligible patients met the following 4 criteria: (1) age 6 months to 18 years at disease onset; (2) fever for at least 2 weeks that at some point rises to ≥39 °C at least once a day and returns to normal between fever peaks; (3) arthritis in ≥1 joint for at least 10 days; (4) at least one of (a) evanescent erythematous rash, (b) generalized lymphadenopathy, (c) hepatomegaly or splenomegaly, or (d) serositis.

FROST enrolled at all active CARRA Registry sites from September 2016 through December 2019. Patients enrolled in the CARRA Registry with recently diagnosed sJIA according to the above criteria were included in the FROST study. Patients were excluded for active infection (including untreated latent tuberculosis), malignancy, or immunization with live virus vaccines within the past 4 weeks. Patients were intended to be untreated for sJIA at the time of FROST enrollment, but prior treatment with non-steroidal anti-inflammatory drugs of unlimited duration or short-term GC use (up to 14 days of oral GC and/or 3 high-dose pulses of intravenous GC) were allowed. To increase the inclusion of patients who were otherwise eligible for the study, patients were included up to 72 hours after initiating a CTP. Patients were excluded if they had MAS (or other severe disease manifestations) at onset that precluded treatment with one of the CTP arms according to the judgement of the treating physician.

**Fig. 1:**
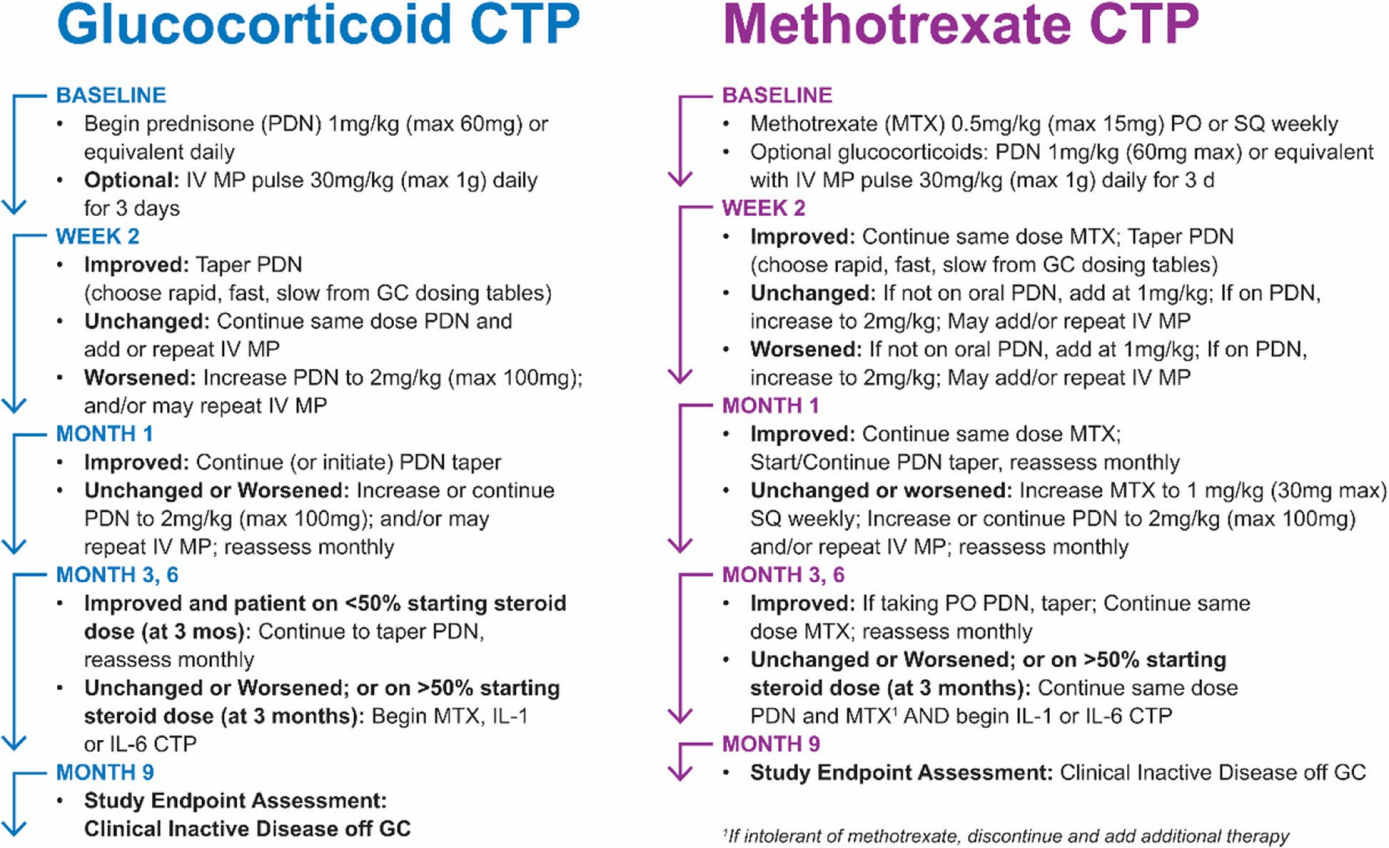
Schematic of the non-biologic consensus treatment plans for the treatment of new-onset sJIA

**Fig. 2:**
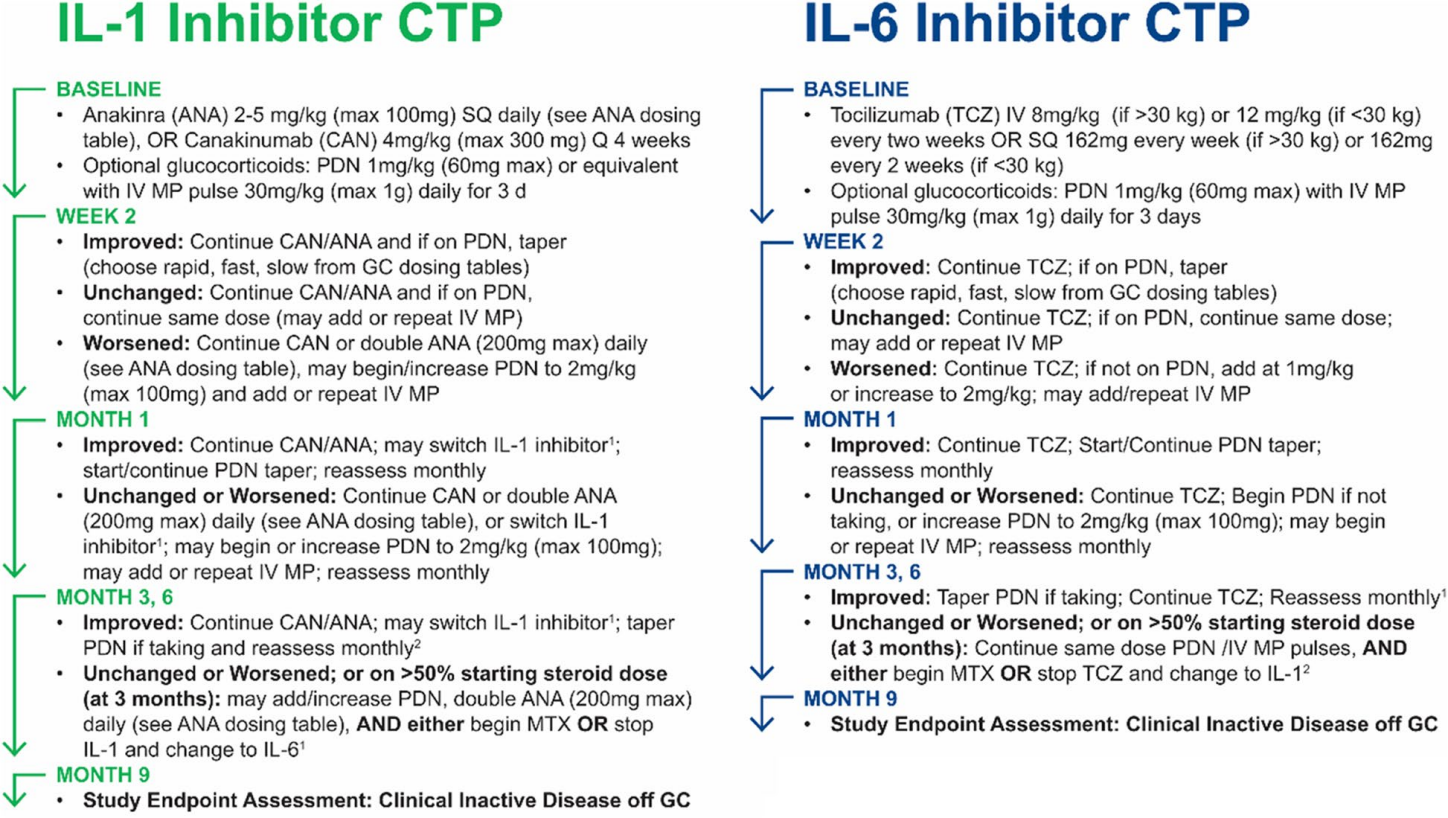
Schematic of the biologic consensus treatment plans for the treatment of new-onset sJIA

In all cases, CTP selection was made by the treating physician in consultation with the patient’s family. Details about the reasons for CTP selection were collected. The 4 CARRA sJIA CTPs have been previously published [14, 20]. In brief, they consist of 2 biologic CTPs (IL-1i and IL-6i, both with or without GC), and 2 non-biologic CTPs (MTX with or without GC and GC alone) (see Figs. 1 and 2). MTX was not included in the biologic CTPs but could be added if patients failed to improve or worsened. For all CTPs, if GC were used, then the stated goal was to reduce the initial GC dose by at least 50% by 3 months and to discontinue GC by 6 months, if possible. For all CTPs, an assessment of the clinical status was to occur 3 months following enrollment; in instances where disease activity was unchanged or worsened or GC could not be safely decreased to < 50% of the initial dose, then the CTPs suggest initiating or switching to a different biologic. Initiation or switching of biologics could also occur at any point during the study at the discretion of the treating physician.

Clinical data were collected at baseline and 2 weeks as well as 1, 3, 6, 9, and 12 months following enrollment. Informed consent and data collection activities for FROST followed the CARRA Registry protocol (Duke University IRB [Pro00054616]). Written informed consent and/or assent was obtained from all subjects and/or their legal guardians.

The primary study outcome was the achievement of clinical inactive disease (CID) according to the Wallace/American College of Rheumatology provisional criteria [21] without current GC use as assessed at 9 months following enrollment. CID criteria include satisfaction of all of the following: (1) no active arthritis; (2) physician global assessment equal to zero; (3) ESR and/or CRP in normal range; (4) no extra-articular features of sJIA; (5) no active uveitis; (6) duration of morning stiffness ≤15 minutes. Not all patients had ESR or CRP values available at the 9 month visit; if the remaining 5 criteria were satisfied, then it was assumed that the patient had achieved CID.

Secondary outcomes included the clinical juvenile arthritis disease activity score based on 10 joints (cJADAS-10) and absence of current GC use. The cJADAS-10 is composed of the physician global assessment (0-10), the patient/parent global assessment (0-10), and the number of joints with active arthritis (to a maximum of 10) [22]. The values of the three components are added together for a total score ranging from 0 to 30. cJADAS-10 scores of ≤1 and ≤ 2.5 have been previously proposed as cut-offs for inactive and low/minimal disease activity for polyarticular JIA, respectively [23], and we additionally required the absence of fever. CID and cJADAS-10 outcomes also were assessed at 12 months after enrollment. Current systemic GC use was assessed at each study visit and is reported as both an independent outcome and in combination with CID and cJADAS-10. We also assessed the proportion of patients receiving oral GC in the first month after enrolment who were able to successfully decrease their dose by > 50% by 3 months after enrollment.

The proportions of patients achieving inactive disease by CID and cJADAS-10, both without current use of GC and irrespective of (i.e., with or without) current GC use were calculated. The results were presented according to the CTP declared at enrollment, irrespective of subsequent treatment (i.e., intention-to-treat). Due to the small numbers of patients in the non-biologic CTPs, no statistical comparisons were made between the CTPs.

Pre-specified safety events of special interest [24], including MAS, were collected for all patients following enrollment. Other safety events were collected if they met the definition of serious adverse events.

## Results

Overall, 73 patients enrolled in the FROST study from 32 clinical sites. Their baseline characteristics are shown in Table 1. Most patients (63/73, 86%) were enrolled in the one of the biologic CTPs. Most patients enrolled early in the disease course. The mean number of days since symptom onset was 46. The median number of days since diagnosis was 2.0, and 75% of patients enrolled within 8 days of sJIA diagnosis. No association was found between patient age, sex, or race/ethnicity with the elapsed time from symptom onset or diagnosis to study enrollment. The mean physician and patient global assessments were 6.0 and 5.6, respectively. The mean number of active joints was numerically lower among patients in the non-biologic CTPs (4.1) than patients in the biologic CTPs (7.0). The median ferritin was also numerically lower among patients in the non-biologic CTPs (363 versus 884). Consistent with study inclusion criteria, all patients had fever and arthritis prior to enrollment. Rash was present prior to enrollment in nearly all patients (95.9%) with other disease manifestations occurring less frequently but relatively equally among patients in the biologic and non-biologic CTPs. Laboratory values at the time of enrollment were consistent with ongoing systemic inflammation.

**Table 1:** Baseline patient characteristics, overall and stratified by consensus treatment plan choice

Characteristic	All Patients	Biologic CTP IL-1i/IL-6i	Non-Biologic CTP GC/Methotrexate
Number of Patients	73	63	10
Age in years (median (IQR))	6.8 (4.1, 11.0)	7.0 (4.0, 11.3)	6.2 (5.6, 7.8)
Male sex (%)	44 (60.3)	40 (63.5)	4 (40.0)
Patient-Reported Race/Ethnicity^a^			
White (%)	48 (65.8)	43 (68.3)	5 (50.0)
Black (%)	7 (9.6)	5 (7.9)	2 (20.0)
Hispanic (%)	14 (19.2)	11 (17.5)	3 (30.0)
Asian (%)	6 (8.2)	6 (9.5)	0 (0.0)
Days since symptom onset (mean (SD))	46.4 (63.5)	49.3 (67.6)	28.1 (18.3)
Days since diagnosis (median (IQR))	2.0 (0.0, 8.0)	1.0 (0.0, 8.0)	5.5 (0.5, 7.0)
Physician global assessment (mean (SD))	6.0 (2.2)	6.3 (2.1)	4.7 (2.8)
Patient global assessment (mean (SD))	5.6 (3.3)	5.7 (3.3)	5.4 (3.6)
Number of active joints (mean (SD))	6.6 (7.6)	7.0 (8.0)	4.1 (4.4)
sJIA manifestations prior to enrollment			
Fever (%)	73 (100.0)	63 (100.0)	10 (100.0)
Arthritis (%)	73 (100.0)	63 (100.0)	10 (100.0)
Rash (%)	70 (95.9)	60 (95.2)	10 (100.0)
Lymphadenopathy (%)	24 (32.9)	22 (34.9)	2 (20.0)
Hepatomegaly or Splenomegaly (%)	15 (20.5)	13 (20.6)	2 (20.0)
Serositis (%)	7 (9.6)	6 (9.5)	1 (10.0)
Laboratory values at time of enrolment (median (IQR))			
ESR (mm/hr)	73 (57, 97)	71 (54, 97)	88 (76, 90)
CRP (mg/L)	15.4 (7.5, 58.1)	16.4 (7.5, 58.1)	13.5 (7.3, 51.4)
Ferritin (ng/mL)	829 (249, 2603)	884 (290, 2652)	363 (81, 779)
Hemoglobin (g/dL)	10.2 (9.1, 11.4)	10.7 (9.1, 11.5)	9.4 (9.0, 10.2)
White blood cell count (10^9^/L)	12.2 (8.5, 19.1)	12.0 (8.4, 19.0)	14.5 (10.6, 22.7)
Platelets (10^9^/L)	458 (353, 571)	452 (353, 565)	509 (375, 735)
CHAQ (mean (SD))	1.3 (1.0)	1.4 (1.0)	1.2 (0.9)
cJADAS-10 (median (IQR))	17.0 (10.5, 21.5)	17.5 (12.0, 21.0)	14.0 (8.0, 23.0)

^a^More than 1 race or ethnicity per patient could be reported

Of the patients enrolled in the biologic CTPs, 59 (94%) were treated with IL-1i and 4 (6%) were treated with IL-6i. Among the 59 initial IL-1i users, the first IL-1i used was anakinra in 48 patients (81%) and canakinumab in 11 patients (19%). During follow-up, 8 (14%) patients initially treated with IL-1i switched to IL-6i treatment; the reason for switching was lack of effectiveness in 2 patients (both receiving anakinra) and their active joint counts near the time of switching were 2 and 8. No patients switched from IL-6i to IL-1i. Nine patients in the biologic CTPs (14%) also started MTX prior to the 9-month outcome assessment.

Among patients in the biologic CTPs, 75% started biologic therapy within 1 day of enrollment and 95% started within 15 days. In addition, 5 (50%) of the patients in the non-biologic CTPs started biologic therapy (3 canakinumab, 1 anakinra, 1 tocilizumab). The elapsed time from enrollment to biologic initiation in the non-biologic CTP patients was 10, 24, 30, 90, and 101 days.

CTP choice appeared to be strongly influenced by physician factors. Ten clinical sites enrolled 3 or more patients in the study. Of these 10 sites, 8 sites enrolled all their patients in the biologic CTPs. Eight of the 10 (80%) patients in the non-biologic CTPs were treated by physicians who self-reported that they initiate treatment with a biologic agent at the time of diagnosis for a typical patient with sJIA of moderate severity less than 50% of the time. On the other hand, only 4% of patients (2 of 56) treated by physicians who self-reported that they initiate treatment of sJIA with a biologic agent at least 75% of the time were enrolled in the non-biologic CTPs.

The three most commonly reported reasons for selecting the biologic CTPs were likelihood of effectiveness for systemic features, minimization of systemic glucocorticoids, and likelihood of effectiveness for arthritis. Two of the three most commonly reported reasons for selecting the non-biologic CTPs were similar citing likelihood of effectiveness for systemic features and likelihood of effectiveness specifically for arthritis; however, safety profile was also included.

More than one-half (43/73, 59%) of patients overall received oral GC at any time during the first month after enrollment, including 34/63 (54%) of patients in the biologic CTPs and 9/10 (90%) in the non-biologic CTPs. At the 3 month assessment, 25/34 (74%) of patients in the biologic CTPs and 5/9 (56%) in the non-biologic CTPs treated with GC, had reduced the GC dose by 50% or more.

Table 2 summarizes the clinical outcomes 9 and 12 months after study enrollment. Data were available for 57 patients at 9 months (16 patients did not have a 9 month visit recorded). Overall, 57% of patients met the primary outcome of CID without current GC use, and 75% had cJADAS-10 scores ≤2.5 with no fever and no current GC use. Patients in the biologic and non-biologic CTPs had similar outcomes, although 4 of the 6 (67%) patients evaluable for CID in the non-biologic CTP had initiated biologics during the study. Outcomes at 12 months were highly similar to the 9 month outcomes (Table 2). Of the patients in the biologic CTPs who subsequently started MTX, 1 of 6 (17%) had CID without concurrent GC use at 9 months. Of the patients in the biologic CTPs who switched from IL-1i to IL-6i, 1 of 6 (17%) had CID without concurrent GC use at 9 months.

**Table 2:** Clinical outcomes at 9 and 12 months following study enrollment

Outcome	All Patients	Biologic CTPs	Non-biologic CTPs
9 months following study enrollment:			
CID without current GC use (N (%))	30/53 (57%)	27/47 (57%)	3/6 (50%)
CID irrespective of current GC use (N (%))	32/53 (60%)	29/47 (62%)	3/6 (50%)
cJADAS-10 ≤ 1 + no fever without current GC use (N (%))	32/48 (67%)	29/43 (67%)	3/5 (60%)
cJADAS-10 ≤ 1 + no fever irrespective of current GC use (N (%))	34/48 (71%)	31/43 (72%)	3/5 (60%)
cJADAS-10 ≤ 2.5 + no fever without current GC use (N (%))	36/48 (75%)	33/43 (77%)	3/5 (60%)
cJADAS-10 ≤ 2.5 + no fever irrespective of current GC use (N (%))	38/48 (79%)	35/43 (81%)	3/5 (60%)
cJADAS-10 (mean (SD))	1.5 (3.3)	1.3 (3.0)	3.4 (5.6)
cJADAS-10 (median (IQR))	0 (0, 1.0)	0 (0, 1.0)	0 (0, 4.0)
12 months following study enrollment:			
CID without current GC use (N (%))	31/55 (56%)	28/49 (57%)	3/6 (50%)
CID irrespective of current GC use (N (%))	34/55 (62%)	30/49 (61%)	4/6 (67%)
cJADAS-10 ≤ 1 + no fever without current GC use (N (%))	33/47 (70%)	31/42 (74%)	2/5 (40%)
cJADAS-10 ≤ 1 + no fever irrespective of current GC use (N (%))	35/45 (78%)	32/40 (80%)	3/5 (60%)
cJADAS-10 ≤ 2.5 + no fever without current GC use (N (%))	36/47 (77%)	33/42 (79%)	3/5 (60%)
cJADAS-10 ≤ 2.5 + no fever irrespective of current GC use (N (%))	38/45 (84%)	34/40 (85%)	4/5 (80%)
cJADAS-10 (mean (SD))	2.0 (5.7)	1.7 (5.4)	4.0 (7.9)
cJADAS-10 (median (IQR))	0 (0, 0.5)	0 (0, 0.5)	0 (0, 2.0)

**Fig. 3:**
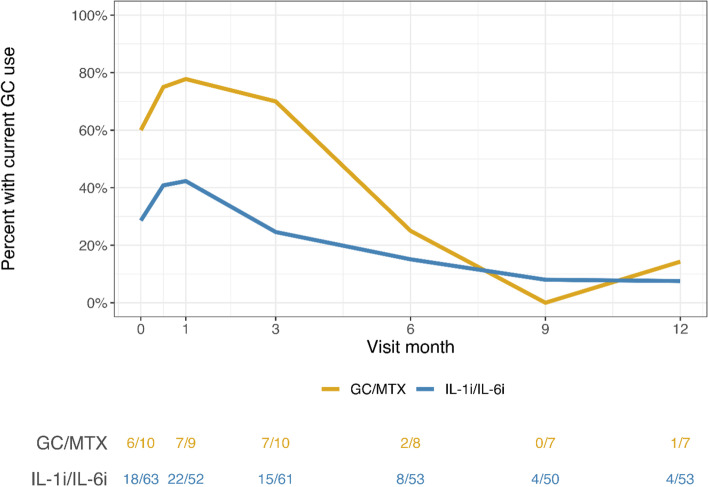
Proportion of patients with current glucocorticoid use at each study visit

Figure 3 shows the proportions of patients receiving current GC at each study visit, stratified by biologic and non-biologic CTP. At 1 month following study enrollment, 22 of 52 (42%) patients treated with biologic CTPs were receiving GC, and 7 of 9 (78%) patients initially treated with non-biologic CTPs were receiving GC. At 6 months following study enrollment, 8 of 53 (15%) patients treated with biologic CTPs were receiving GC, and 2 of 8 (25%) patients initially treated with non-biologic CTPs were receiving GC. At 9 and 12 months following study enrollment, the proportion of patients receiving GC was less than 15% in both biologic and non-biologic CTPs.

Overall, there were 16 CTCAE grade 3 or higher safety events observed in 13 patients during follow-up, and all of these events occurred in patients in the biologic CTPs. Table 3 lists the events and the current biologic and non-biologic medications at the time of the safety event. One patient treated with a biologic CTP died 2.6 years after study enrollment of acute liver failure in the absence clinical signs of MAS or drug reaction with eosinophilia and systemic symptoms (DRESS).

**Table 3:** Biologic and non-biologic medication use at the time of safety events with CTCAE grade 3 or higher

Event	CTCAE grade	CTP Arm	Current Biologic and Non-Biologic Use
Acute liver failure	5	Biologic	anakinra
Liver enzyme elevation	4	Biologic	anakinra
MAS	4	Biologic	anakinra
Injection site reaction	3	Biologic	anakinra
Infection (osteomyelitis)	3	Biologic	canakinumab
Liver enzyme elevation	3	Biologic	anakinra
MAS	3	Biologic	canakinumab
MAS	3	Biologic	canakinumab
MAS	3	Biologic	canakinumab
MAS	3	Biologic	none
MAS	3	Biologic	none
Neutropenia	3	Biologic	tocilizumab
Neutropenia	3	Biologic	anakinra
Protein losing enteropathy	3	Biologic	anakinra
SJIA flare	3	Biologic	canakinumab
SJIA flare	3	Biologic	anakinra, cyclosporine

## Discussion

The FROST study prospectively enrolled a large cohort of patients with new-onset sJIA treated with one of four CTPs from 2016 through 2019 in the CARRA Registry. Most patients were treated with biologics (mostly IL-1i) and achieved the primary endpoint of CID off GC at 9 and 12 months. Seventy-five percent of patients achieved a cJADAS-10 of <=2.5 (cJADAS-10 “inactive disease” for polyarticular JIA) without fever and GC use. The original goal of the study was to compare the effectiveness of starting a biologic CTP (IL-1i or IL-6i) vs a non-biologic CTP (MTX or GC alone) using propensity scores to create balance between CTP groups at baseline and Bayesian methods that incorporated prior expert opinions [25]. This was not possible because too few patients started on a non-biologic CTP. In addition, 50% of the patients starting a non-biologic CTP initiated a biologic by the 3 month visit, making the comparison of outcomes at 9 months less meaningful. However, despite these shortcomings, the outcomes at 9 and 12 months demonstrate that most patients in the current era with sJIA, a previously difficult to treat disease, are faring well.

Our results, which showed that more than 50% of patients achieved CID off GC and 75% of patients achieved cJADAS10 inactive disease status at 9 months, generally align with previous studies of early biologic use in sJIA. The initial study assessing the impact of anakinra treatment on sJIA was a 2011 retrospective case series of 46 patients from multiple centers treated with anakinra as part of initial therapy [4], found that at 6 or more months after starting anakinra, 89% did not have active arthritis, and 60% had a “complete response.” The prior study results appear better at first glance, but it was a retrospective study that was unable to assess CID status or JADAS scores. A more recent prospective Treat to Target study of 42 patients treated with anakinra in the Netherlands and published in 2019, showed that 76% had inactive disease at 1 year with 52% in inactive disease off all medications (including anakinra) [9]. Impressively, after up to 5 years of follow up, less than 5% reported joint damage and only 33% were ever treated with GC. A third study published in 2021 of 56 patients treated with anakinra showed that 73% had achieved CID off GC at 6 months, and that patients treated prior to 3 months disease duration had a better outcome [26].

Together, current and prior studies suggest a potential window of opportunity for new onset sJIA patients: early treatment with biologics (IL-1i but potentially other biologics as well), can lead to rapid disease control associated with better long-term outcomes. The pathophysiological basis for such a window remains incompletely defined, but has been postulated to reflect the efficacy of early cytokine antagonist therapy to abrogate the development of a population of arthritis-causing T cells, a possibility for which experimental evidence has begun to accumulate [8, 27, 28]. Previously it was commonly reported that sJIA patients developed chronic relapsing systemic disease with recurrent MAS and/or chronic, often severe and debilitating arthritis that necessitated early joint replacement, although these reports may have been subject to selection bias. With early effective treatment the disease phenotype indeed appears to be altered in many patients, some of whom appear to go into remission without further need for medications, and/or never develop the chronic arthritis phenotype. It is worth mentioning that a minority of sJIA patients remit spontaneously in the first year of disease, complicating the interpretation of single-arm observational studies [29].

A potential downside to early use of biologics may be the development of chronic and often fatal lung disease in sJIA patients which has only recently been described. While a rare phenomenon, chronic lung disease appears to be occurring with increased frequency since 2005, raising the possibility that early treatment with biologics could be at least partially responsible [10–13]. It is noteworthy that no patient in the present study developed this complication.

The early pilot study results of the sJIA CTPs indicated there was a reasonable distribution of CTP usage that appeared to be related to specific sites, with some sites preferring non-biologic CTPs while others preferred biologic CTPs, raising the possibility of “pseudo-randomization” (i.e., approximating cluster randomization by clinical site) might be possible in a larger observational study [16, 25]. Pseudo-randomization in FROST by physician preference was observed (i.e., some sites nearly always initially treated patients with biologics, irrespective of the clinical circumstances). Nevertheless, most clinicians preferred starting with a biologic CTP (especially with IL-1i) by the time this study began, reflecting a shift in clinical practice, and making the original aim of a comparative effectiveness study infeasible. There would likely have been a more balanced distribution of CTP use with more patients receiving non-biologic CTPs if this study had been performed soon after the development of the original CTPs. However, since the original intent of CARRA CTP development was to standardize community treatments, eliminating unsuccessful or unused treatments, the next iteration of the sJIA CTPs will consider the treatment preferences and results observed in FROST when determining the treatment arms. Moreover, randomization of patients, even if open-label, would have not been feasible or ethical in this population of patients with a rare illness in which practice has dramatically changed due to the availability of effective treatments.

It remains a limitation of this study, however, that there was no randomization which may have resulted in confounding by treatment choice. The relative availability of different biologics can vary by country (due to cost, regulatory approval, etc.), and the treatment choices in this study may not be generalizable to all regions outside of the United States and Canada. There were missing data on some components of the various study outcomes and some missed study visits that limited the assessment of outcomes. Notably, there was difficulty enrolling patients even with the modified sJIA criteria; many providers were reportedly unwilling to delay potentially efficacious treatment in patients suspected of having sJIA even if they did not fulfill all FROST criteria, especially arthritis. Despite the inclusion of sJIA in the disease classification of JIA, not all sJIA patients develop arthritis, and is not a requirement in the Yamaguchi criteria for AOSD [30] or the proposed PRINTO classification criteria [31]. Lastly, we were unable to use the new systemic JADAS [32] as a measure of disease activity because the precise body temperatures (required for scoring) were not collected. Instead, we used the cJADAS10 with the addition of absence of fever.

## Conclusions

It is strongly encouraging that the majority of patients with new onset sJIA had excellent outcomes, with less GC usage than was necessary prior to the availability of biologics. The availability of biologics effective for treating sJIA has undoubtedly changed outcomes for the vast majority of patients with this disease. We look forward to following the outcomes of these patients in the longer term, since all FROST patients are enrolled in the CARRA Registry, enabling follow up for at least 10 years.

## Data Availability

The data that support the findings of this study are available from CARRA but restrictions apply to the availability of these data, which were used under a data use agreement for the current study, and so are not publicly available. Data are however available from CARRA upon reasonable request (carragroup.org).

## Abbreviations

AOSD: Adult-onset Still disease
CARRA: Childhood Arthritis and Rheumatology Research Alliance
CHAQ: Childhood health assessment questionnaire
CID: Clinical inactive disease
cJADAS-10: Clinical juvenile arthritis disease activity score based on 10 joints
CRP: C-reactive protein
CTP: Consensus treatment plan
ESR: Erythrocyte sedimentation rate
FROST: First-line options for sJIA treatment
GC: Glucocorticoids
IL-1i: IL-1 inhibitor
IL-6i: IL-6 inhibitor
IQR: Interquartile range
JIA: Juvenile idiopathic arthritis
sJIA: Systemic juvenile idiopathic arthritis
MAS: Macrophage activation syndrome
MTX: Methotrexate
SD: Standard deviation
STOP-JIA: Start time optimization of biologics in polyarticular JIA
WBC: White blood cell count
